# Paget's disease with tumefactive soft tissue extension mimicking a chronic subdural hematoma

**DOI:** 10.1016/j.radcr.2024.04.076

**Published:** 2024-05-17

**Authors:** Khadija Soufi, Omar Ortuno, Gabriel Urreola, Jose Castillo

**Affiliations:** Department of Neurological Surgery, University of California, Davis, Medical Center, 4301 X St, Sacramento, CA 95817, USA

**Keywords:** Paget's, Sclerosis, Hemicalvarium, Tumefactive, Bone, Skull

## Abstract

Paget's disease, the leading cause of skull sclerosis, is often under-diagnosed on imaging with tumefactive soft issue extension being mistaken for other intracranial findings. A 71-year-old female with past medical history of hypertension, chronic obstructive pulmonary disease, transient ischemic attack, 7 pack year smoke history, and alcohol abuse experienced an episode of bilateral upper extremity weakness, left arm numbness, left hand clumsiness, and word deficits that resolved within 20 minutes. Head computed tomography showed a right convexity mass measuring 6 mm with slight mass effect on the right cerebral hemisphere but no midline shift. She also had a sclerotic calvarium with focal erosions, periosteal reaction and scalp edema with no evidence of acute infarct, significant stenosis, occlusion, and aneurysm of the major intracranial arteries. Additional magnetic resonance imaging was ordered. The pattern of sclerosis of the right hemicalvarium extending into the left hemicalvarium and areas of abnormal bony texture and enhancement where sclerosis had not occurred suggested this to be the sclerotic phase of Paget's Disease. Additionally, the enhancing soft tissues on either side of the right hemicalvarium and overlying the posterior left parietal bone were thought to represent benign tumefactive soft tissue or pseudotumor. Tumefactive lesions often present a differential dilemma that is best resolved through a multi-disciplinary approach with extensive review on clinical and imaging findings. Tumefactive soft tissue extension related to Paget's disease of the skull has not been described in the literature and our case study highlights the importance of considering this entity on one's differential for patients presenting with an extra-axial lesion.

## Introduction

Paget's disease (PD) is the leading cause of skull sclerosis, a process that consists of multiphasic, abnormal, and excessive bone turnover causing remodeling and distortion of the underlying bony architecture [[7], [8], [9]]. This process involves 3 histological phases: a lytic phase with bone resorption, followed by a mixed phase of bone resorption and deposition, and finally a sclerotic phase. Although findings are typically limited to the skull, PD has been associated with enhancing tumefactive soft tissue extension which can be mistaken for a variety of intra-cranial findings [[1], [2], [3], [4], [5], [6]]. Thus, the diagnosis of PD with appropriate imaging and clinical correlation avoids unnecessary biopsy and surgical intervention resulting in prompt treatment. Given the complexity and rarity of these findings, PD is often under-diagnosed on imaging [[1], [2], [3], [4], [5], [6]].

We report the first case of PD of the skull with tumefactive soft tissue extension in a patient presenting with neurological symptoms related to local mass affect, and normal laboratory findings. A literature review of PD cases and diagnostic approaches is also discussed.

## Illustrative case

A 71-year-old female with past medical history of hypertension, chronic obstructive pulmonary disease, and transient ischemic attack (TIA) awoke following a diagnostic esophagogastroduodenoscopy for gastroesophageal reflux disease (GERD) symptoms with a brief episode of bilateral upper extremity weakness with significant clumsiness of her left hand, left extremity numbness and word deficits. She had complete resolution of her symptoms within 20 minutes. Additional findings included mild recurrent headaches over the last couple years and firm nodules on her scalp that had been previously discussed with her primary care providers and being visually monitored. She denied any history of vision or hearing changes, nausea, vomiting or seizure but did endorse 7 pack year smoking history and alcohol abuse.

**Fig. 1:**
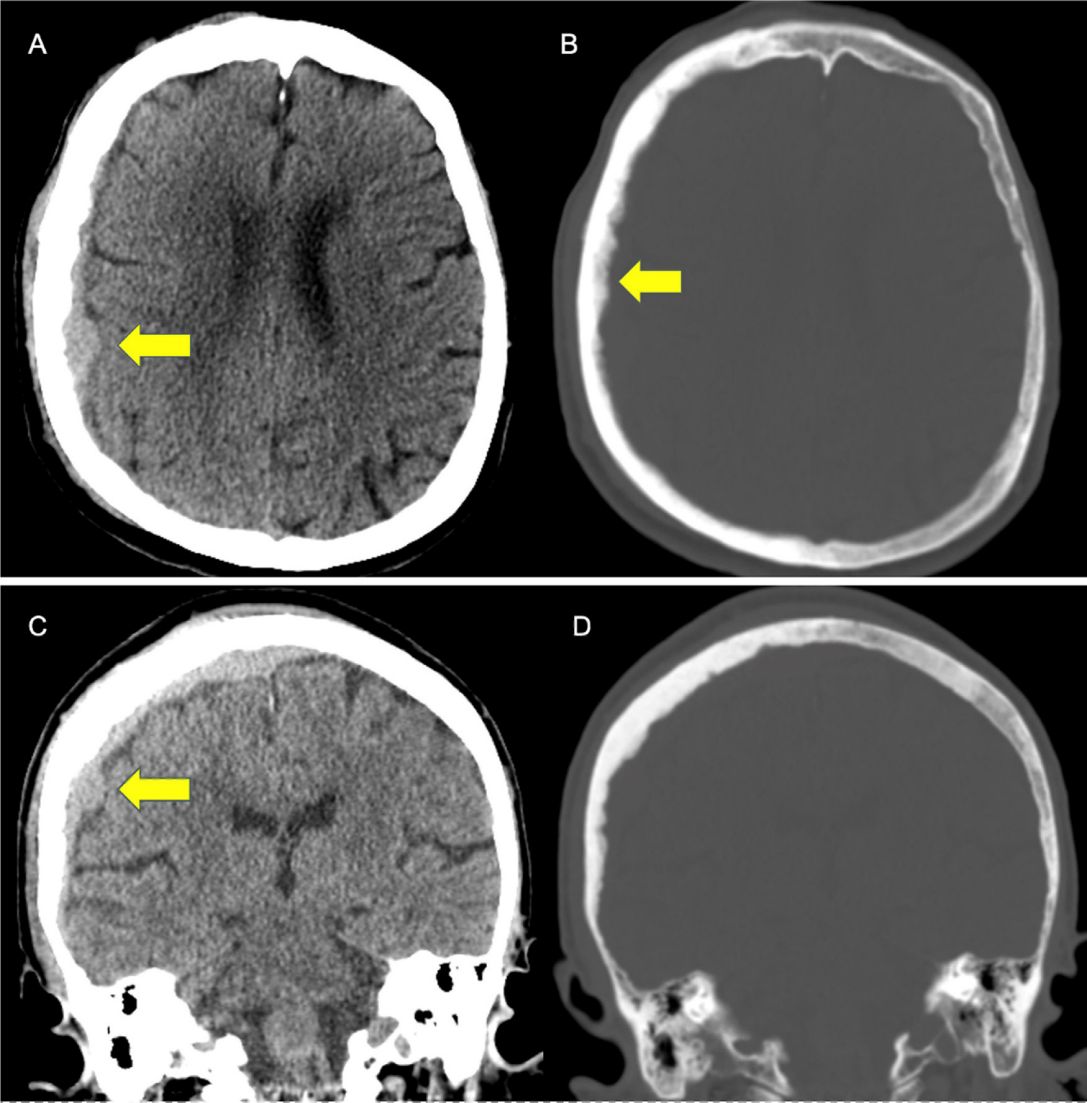
Computed tomography images of the head, axial, and coronal views. (A and C) Isodense subdural collection on the right (yellow arrow). (B and D) Bone view showing a hyperdense right skull when compared to the left along with subtle, hypodense portion near the inner cortex (yellow arrow).

Given her history of prior TIA and similar re-occurrence of her symptoms, she underwent head and neck computed tomography (CT) angiogram to further evaluate symptoms. On CT of the head she was found to have a right convexity mass measuring 6 mm with slight mass effect on the right cerebral hemisphere but no midline shift (Fig. 1). Additional findings included a sclerotic calvarium with focal erosions, periosteal reaction, and scalp edema with no evidence of acute infarct, significant stenosis, occlusion, and aneurysm of the major intracranial arteries. Her findings were initially thought to be compatible with a chronic subdural hematoma (SDH), with a differential diagnosis that included primary or secondary neoplasm.

**Fig. 2:**
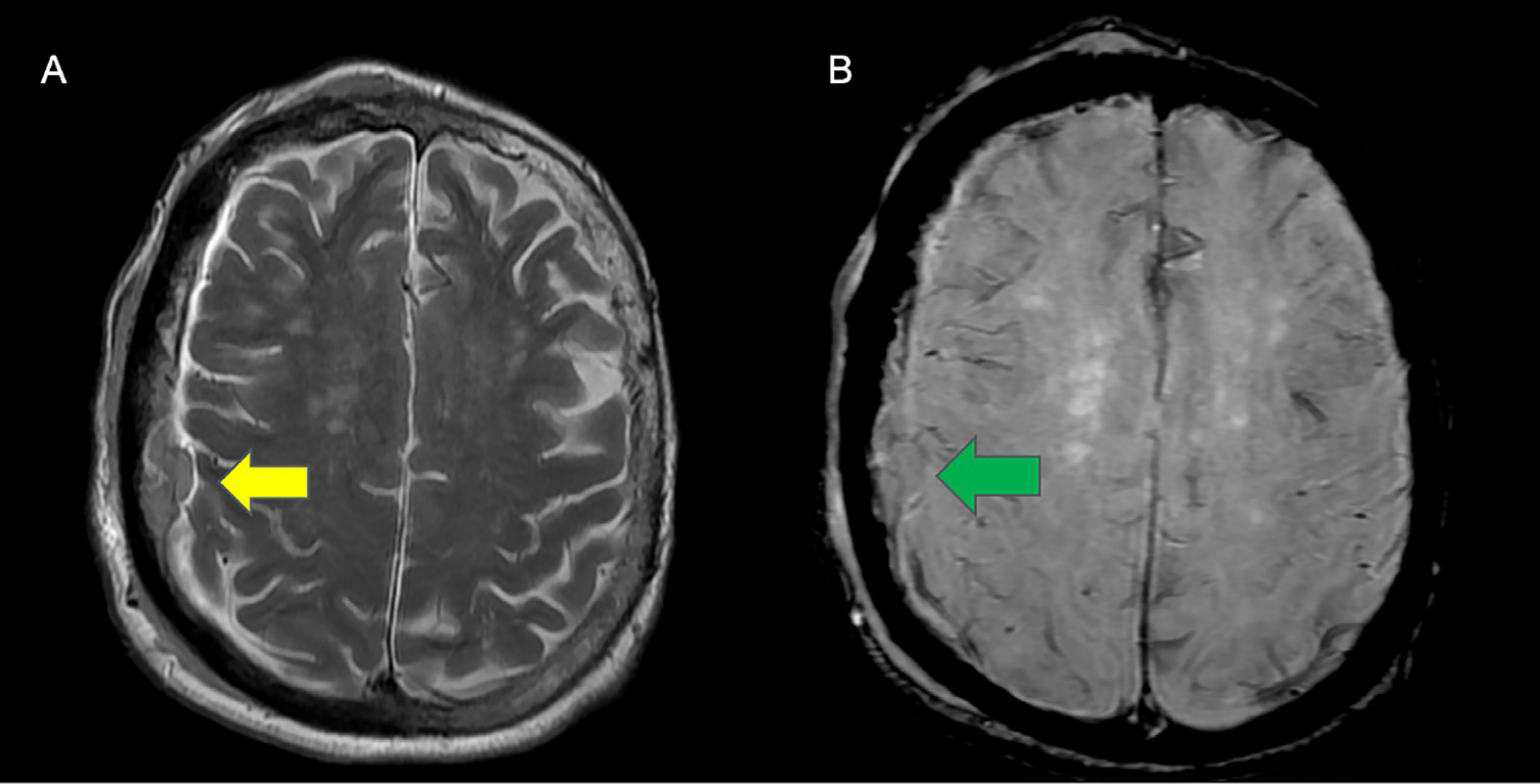
Magnetic resonance imaging of the brain, axial views. (A) T2 sequence showing a hypointense right sided subdural collection (yellow arrow). (B) SWI sequence showing no blood products withing the right sided subdural collection (green arrow).

**Fig. 3:**
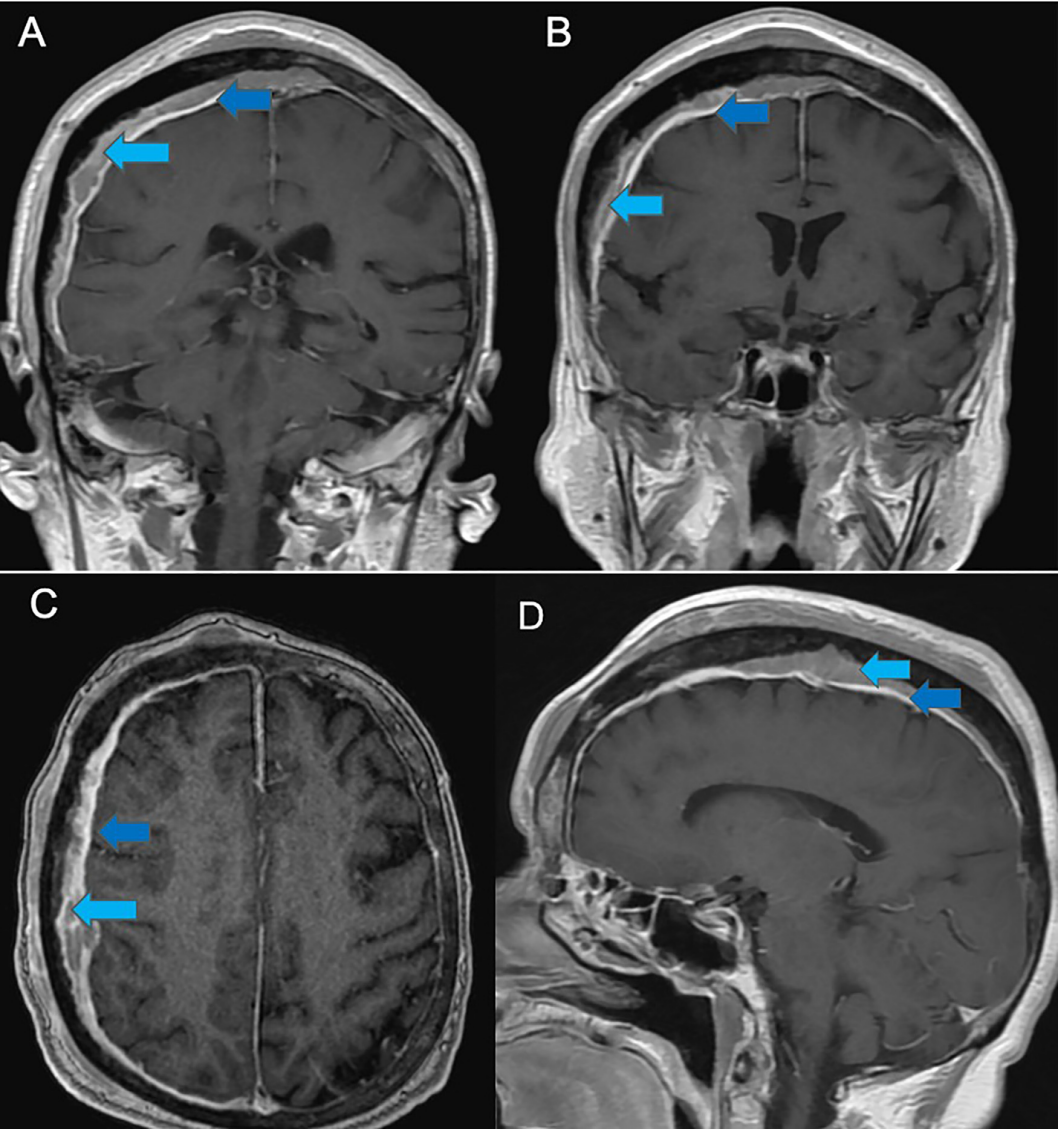
Magnetic resonance imaging of the brain, T1 sequences post contrast administration, coronal, axial, and sagittal views. (A and B) are coronal views showing near homogenous enhancement of the right subdural collection (light blue arrow) with underlying dural enhancement (dark blue arrow). (C and D) are axial and sagittal views, respectively, showing similarly.

Following transfer to our institution, a magnetic resonance imaging of the brain (Fig. 2, Fig. 3) was obtained followed by a review of the imaging findings by a multi-disciplinary team of neurosurgeons, neuroradiologists, and oncologists. Pertinent discussions focused on the pattern of sclerosis of the right hemicalvarium extending into the left hemicalvarium and areas of abnormal bony texture and enhancement where sclerosis had not occurred. These radiographic findings were most compatible with the sclerotic phase of PD. Additionally, the enhancing soft tissues on either side of the right hemicalvarium and overlying the posterior left parietal bone were thought to represent benign tumefactive soft tissue or pseudotumor and not an SDH.

**Fig. 4:**
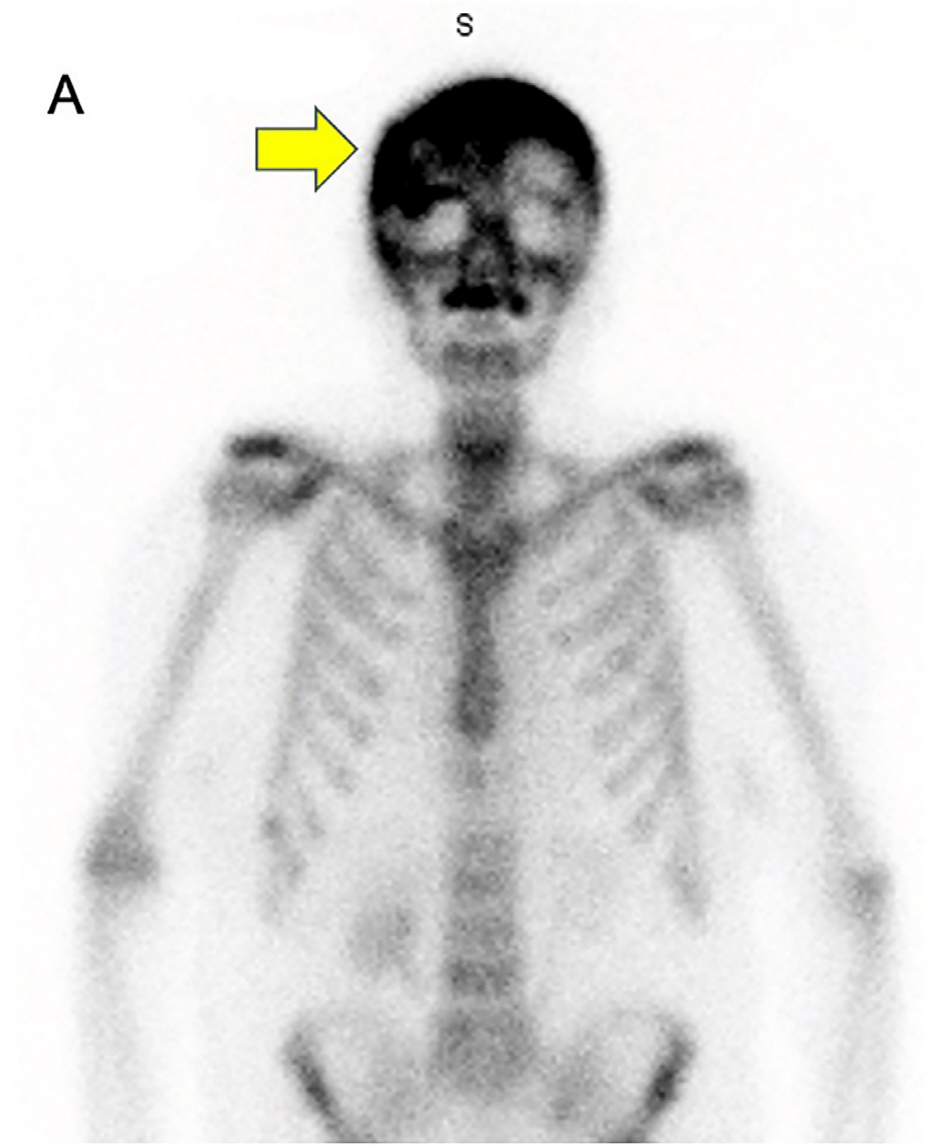
Whole body bone scintigraphy, anterior view. (A) Increased radiotracer uptake in the cranium, especially on the right frontal prominence that coincides with the lucent area found on CT (yellow arrow).

The patient was started on Keppra prophylaxis and routine electroencephalogram monitoring given possible concern for seizure activity since the patient was amnestic to the event and had bilateral abnormal movements. On further questioning the patient endorsed tightening of her glasses overtime and a 20 pound unintentional weight loss in the last year. Her laboratory findings consisted of elevated erythrocyte sedimentation rate of 34 mm/hr (reference 0-30 mm/hr), low blood calcium of 1.12 mmol/L (reference 1.17–1.31 mmol/L), normal phosphorous levels of 3.3 mg/dL (reference 2.5–4.5 mg/dL), alkaline phosphatase (ALP) of 85 U/L (reference 35–129 U/L), bone specific ALP of 10.9 ug/L (reference in premenopausal females 4.5–16.5 ug/L; reference in postmenopausal females 7.0–22.4 ug/L), parathyroid hormone of 23 pg/mL (reference 15–65 pg/mL) and vitamin D, 25 hydroxy of 20.7 ng/mL (reference 10–50 ng/mL). Her basic metabolic panel results had been within normal range for the last 1.5 years. Her normal protein and albumin levels lowered the suspicion for multiple myeloma along with absent lytic lesions. Other differentials included metastatic malignancy, osteosarcoma or giant cell tumors of bone and osteomalacia which were unlikely given negative systemic imaging (CT chest/abdomen/pelvis), and her recent weight loss was thought to be related to her severe GERD symptoms and decreased oral intake. The normal ALP suggested that the patient was in the early sclerotic stage of PD and was further supported by the radiographic findings of sclerotic lesions. Given her stable condition and low likelihood of malignancy, the patient was discharged, and a follow-up radionuclide bone scan (Fig. 4) showed increased metabolism of the skull. The patient was referred for endocrinology follow-up and was thereafter lost to follow-up.

## Discussion

The clinical signs and symptoms of extra-axial lesions with local mass effect are often vague and nonspecific. In some instances, patients may report a prolonged asymptomatic lucid period followed by progressively worsening localizing neurological symptoms. In other cases, patients present with acute neurological deterioration. Hence, a timely diagnosis prior to clinical deterioration is crucial and reliant on obtaining the appropriate imaging.

The clinical presenting symptoms of patients with extra-axial lesions are related to the location of the lesion, extent of local mass effect and any associated peri-lesional inflammatory response and time course of disease. Common focal neurological signs include hemiparesis in frontal lobe extra-axial lesions, speech or sensory impairment for the parietal lobe, cranial nerve palsy and meningitis at the posterior fossa, as well as falx syndrome for interhemispheric bleeds. However, some patients present with no symptoms especially in cases of chronic SDH.

Common extra-axial lesions include traumatic subdural hematomas which form between the dura and the arachnoid mater overlying the brain and adopts the classic crescent shaped distributions as the bleeding follows the contour of the overlying dura. In the acute setting, patients with progressively worsening unilateral neurological symptoms, with radiographic findings of an extra-axial lesion with local mass effect should undergo urgent surgical evacuation. Extra-axial lesions such as subdural hematomas are secondary to tearing of bridging veins or arterial tears in 20%-30% of patients. Head trauma including motor vehicle accidents, falls, and assaults are the leading cause of SDH. Many patients, however, have no noted history of trauma, especially amongst those with cerebral atrophy, history of intracranial hypotension, of the elderly. In addition, establishing the diagnosis in the elderly can further be complicated by other comorbidities such as a history of malignancy and/or metastatic disease.

The differential diagnosis for extra-axial brain lesions is broad and the definitive diagnosis can be complicated in some cases. Familiarity with a variety of causes for extra-axial brain lesions with awareness of their radiographic findings is essential to improve diagnostic accuracy and optimize patient management. Analyzing the radiographic findings in the context of the clinical presentation allows for a more accurate imaging interpretation and facilitates the management of patients. In this case presentation, the patient's symptoms were non-localizing and transient warranting a more comprehensive work-up and the need for an informed differential diagnosis in the context of the presenting illness.

Tumefactive lesions often present a differential dilemma, and if correctly diagnosed should not be biopsied. Key radiographic features such as paucity of mass-effect or minimal/no perilesional edema may aid in distinguishing some tumefactive lesions from neoplasms. Tumefactive soft tissue extension related to PD of the skull, however, has not been described in the literature. In this case report, assessment of the bone window revealed incidental sclerotic osseous abnormalities of the craniofacial skeleton which was essential to our evaluation. There are multiple causes of sclerotic skull changes including fibrous dysplasia, meningiomas, osteomalacia and wide breath of malignancies including osteosarcomas, multiple myeloma, Hodgkin lymphoma and POEMS (polyneuropathy, organomegaly, endocrinopathy, M-protein, and skin changes) syndrome. Appropriate differentiation between etiologies requires pattern analysis best achieved through a multi-disciplinary approach with extensive review on clinical and imaging findings. Although most of these bone window imaging findings are unrelated to the underlying pathology, symptoms may present due to lesion mass effect or cortical irritation.

PD remains the leading cause of skull sclerosis and consists of multiphasic, abnormal, and excessive bone turnover which results in remodeling of the bone with underlying distorted architecture [[7], [8], [9]]. Histologically, PD consists of three consecutive phases including the lytic phase with bone resorption, followed by a mixed phase of bone resorption and deposition and finally a sclerotic phase. The imaging findings of each stage vary, and the skull has specific unique findings including osteoporosis circumscripta, lytic lesions involving only the inner aspect of the outer, cotton wool appearance, a mixed lytic and sclerotic lesion, diploic widening of both inner and outer calvarial tables, and lastly Tam o' Shanter sign, platybasia and basilar invagination with the appearance of the skull falling over the facial bones. Although, findings are typically limited to bony skull, PD may be associated with enhancing benign tumefactive soft tissue extension which can be mistaken for a variety of intra-cranial findings including a subdural hemorrhage [[1], [2], [3], [4], [5], [6]]. The typical features of tumefactive soft tissue extension include enhancing soft tissues that is the brain can also take on the classic crescentic shape of an extra-axial lesion such as an SDH. Key differentiating factors include lytic and sclerotic skull changes with enhancing soft tissue on both sides of the skull while a SDH would only present with enhancing mass underneath the skull in the acute time period.

Laboratory findings are another important consideration for Paget's disease and there are several indices of increased bone turnover for both monitoring and diagnostic purposes. ALP elevation may be the first and only sign of PD, however, caution must be utilized since ALP may be elevated both due to bone and liver pathologies. In this case study, bone specific ALP was also utilized and is useful in cases of diagnostic uncertainty. Of note, normal levels of ALP may be present despite significant disease burden especially with patients are in the sclerotic stage with limited bone turn-over. Despite elevated bone turnover, calcium and phosphate levels are expected to be within normal range for patients with Paget's disease, however, exceptions are possible in those with extremely active disease.

We present the first case report of PD of the skull with tumefactive soft tissue extension in a patient with transient symptoms and normal laboratory findings. Radiographic findings demonstrated an extra-axial lesion initially suspected to be an SDH, but in the context of the clinical history represented tumefactive soft tissue extension. Herein, we report another manifestation of Paget's disease and highlight the importance of considering this entity on one's differential for patients presenting with an extra-axial lesion.

## Patient consent

The authors certify that they have obtained all appropriate patient consent forms.
